# Psychometric Properties of the Eating Disorder Examination Questionnaire (EDE-Q) and Norms for Rural and Urban Adolescent Males and Females in Mexico

**DOI:** 10.1371/journal.pone.0083245

**Published:** 2013-12-18

**Authors:** Eva Penelo, América Negrete, Mariona Portell, Rosa M. Raich

**Affiliations:** 1 Laboratori d’Estadística Aplicada, Departament de Psicobiologia i Metodologia de les Ciències de la Salut, Universitat Autònoma de Barcelona, Bellaterra (Cerdanyola del Vallès), Barcelona, Spain; 2 Academia Instrumental del Programa Académico de Psicología, Universidad Autónoma de Nayarit, Tepic, Nayarit, Mexico; 3 Unitat d'Avaluació I Intervenció en Imatge Corporal, Universitat Autònoma de Barcelona, Bellaterra (Cerdanyola del Vallès), Barcelona, Spain; 4 Departament de Psicobiologia i Metodologia de les Ciències de la Salut, Universitat Autònoma de Barcelona, Bellaterra (Cerdanyola del Vallès), Barcelona, Spain; 5 Departament de Psicologia Clínica i de la Salut, Universitat Autònoma de Barcelona, Bellaterra (Cerdanyola del Vallès), Barcelona, Spain; Paris Institute of Technology for Life, Food and Environmental Sciences, France

## Abstract

**Aims:**

To contribute new evidence to the controversy about the factor structure of the Eating Disorder Examination Questionnaire (EDE-Q) and to provide, for the first time, norms based on a large adolescent Mexican community sample, regarding sex and area of residence (urban/rural).

**Methods:**

A total of 2928 schoolchildren (1544 females and 1384 males) aged 11-18 were assessed with the EDE-Q and other disordered eating questionnaire measures.

**Results:**

Confirmatory factor analysis of the attitudinal items of the EDE-Q did not support the four theorized subscales, and a two-factor solution, Restraint and Eating-Shape-Weight concern, showed better fit than the other models examined (RMSEA = .054); measurement invariance for this two-factor model across sex and area of residence was found. Satisfactory internal consistency (ω ≥ .80) and two-week test-retest reliability (ICCa ≥ .84; κ ≥ .56), and evidence for convergent validity with external measures was obtained. The highest attitudinal EDE-Q scores were found for urban females and the lowest scores were found for rural males, whereas the occurrence of key eating disorder behavioural features and compensatory behaviours was similar in both areas of residence.

**Conclusions:**

This study reveals satisfactory psychometric properties and provides population norms of the EDE-Q, which may help clinicians and researchers to interpret the EDE-Q scores of adolescents from urban and rural areas in Mexico.

## Introduction

The Eating Disorder Examination Questionnaire (EDE-Q) [1] is a self-report questionnaire that is widely used to measure disordered eating. It was derived from the Eating Disorder Examination interview (EDE) [2], which can be considered the method of choice for assessing the specific psychopathology of eating disorders (ED). The EDE-Q assesses not only eating disorder attitudes, but also specific behaviours -and their frequency- related to ED as defined by the Diagnostic and Statistical Manual of Mental Disorders (DSM-IV) [3]. 

One limitation of the EDE-Q is that the theoretical factor structure of attitudinal items consisting of four subscales (Restraint, Eating concern, Shape concern, and Weight concern) has not been supported empirically. In recent years, several studies in community and ED samples [4-12] (also Waller and Pennings & Wojciechowski, both as cited in Allen et al.) [5] and obese bariatric surgery candidates [13,14] have focused on this issue. None of them has provided evidence of a better fit of the original 4-factor model when compared with other plausible competing models, since it appears that the "concern" items, particularly the Shape concern and Weight concern items, may contribute to a single factor [5]. Considering studies that included all 22 attitudinal items, several modified models have been suggested, mostly due to the fact that Shape and Weight concern items may often show overlap. Firstly, support has been found for three factors approximately corresponding to dietary restraint, eating related concerns, and shape and weight concerns [6,8-12] (also Waller, as cited in Allen et al.) [5]. These 3-factor models have been obtained with exploratory factor analysis (EFA) and non-orthogonal rotation methods (allowing correlated factors) in samples of adult women suffering an ED from England (*N* = 166) (Waller, as cited in Allen et al.) [5] and the United States (*N* = 203) [11], in a community sample of 917 girls and boys from UK [12], and in 1637 healthy American males and females [8], and with principal component analysis (PCA) and varimax rotation (assuming uncorrelated components) in the German adult population (*N* = 2520) [10]. Also, 3-factor models have been evaluated with confirmatory factor analysis (CFA) in 569 English adults [6], 500 Greek undergraduate female students [9], and 1637 American participants [8]. Secondly, due to the excessively high inter-factor correlations found for the original 4-factor model, a 2-factor model (Restraint remaining separated from the three Concern subscales) or a single 1-factor model have been proposed after using EFA in 532 adolescent Fijian females [7]. And thirdly, correlations between items belonging to the same theorized subscale and correlations between items belonging to different theorized subscales have been found to be fairly similar, suggesting that the theorized four subscales are in fact not separable sub-dimensions, but rather that all items together tap into one general underlying dimension [4]. Support for a 1-factor model has been found in samples of Dutch ED patients using CFA (Pennings & Wojciechowski, as cited in Allen et al.) [5] or PCA (*N* = 935) [4]. 

To sum up, EFA has supported the Restraint and Eating concern subscales, whereas most items from the Shape concern and Weight concern subscales have been combined into one factor [10-12] (also Waller, as cited in Allen et al.) [5]. CFA has also supported this 3-factor model [6,8,9], despite the high correlation values of .90 [5] and .84 [6] found between Eating concern and Shape-Weight concern may suggest only a distinct Restraint subscale vs. "concern" items (2-factor model) [7] or even the use of the EDE-Q global summary score as a unitary dimensional construct (1-factor model) [4,7] (also Pennings & Wojciechowski, as cited in Allen et al.) [5].

Regarding the reliability of EDE-Q, acceptable internal consistency has been demonstrated, with Cronbach’s alpha values ranging from .65 to .93 for subscale scores and ≥ .90 for the global score [4,7,9-11,13-21]. Acceptable test-retest reliability has also been demonstrated for attitudinal scores 1 week later (ICC from .55 to .79) [7], 2 weeks later (Pearson *r* from .79 to .94) [15,21], 1-14 days later (Spearman rho from .66 to .93) [19,22], and 141-444 days later (Pearson *r* from .57 to .79) [16]; and for the presence of behavioural features 1 week later (kappa from .39 to .81, except for laxative use with κ = .13) [7], 2 weeks later (phi coefficient from .57 to .70) [15], and 1-14 days later (Spearman rho from .51 to .84) [19,22], but lower 141-444 days later (phi coefficient from .28 to .44) [16] (see also Berg et al.) [23].

In relation to convergent validity, medium to large and statistically significant correlations have been reported between attitudinal EDE-Q scores and related measures assessing similar constructs with the Eating Attitudes Test [9,21,23], Eating Disorder Inventory [18,20,24], and other body image tools [7,13,14,21]. Lastly, few studies have focused on gender differences with EDE-Q. Findings in general population samples have shown that females tend to score higher on attitudinal EDE-Q measures [10,12,17,18,21], and tend to endorse more frequently unhealthy weight-control behaviour such as fasting, use of laxatives and diuretics or self-induced vomiting to lose weight than males [10,17,18], although effect sizes have been found to be small to moderate [10]. And males are more likely than females to make use of frequent exercise as a compensatory behaviour [18], while other studies have not found any differences [10,12,17].

Studies of disordered eating in Mexico are scarce and most of them have focused on metropolitan areas [25]. Due to the geographical proximity to the United States, the country whose culture places extreme value on physical attractiveness [26], it is important for tools to be validated by providing empirical evidence of psychometric properties and norms for the EDE-Q among Mexican adolescents, because of its widespread use. 

We aimed to study the psychometric properties of the Spanish version of EDE-Q in male and female schoolchildren (under 19 years) from the Mexican population in the state of Nayarit. This study intends to tackle both urban and rural areas of Mexico, using a rurality criterion that is not only based on demographic indicators. Nayarit includes municipalities with levels of social backwardness from very low to low (85%) and between high and very high (15%) [27]. Regarding the Gini coefficient of inequality (a relative measurement of economic inequality, which can range from 0 -perfect equality in the distribution of income - to 1 - maximal inequality-), CONEVAL (National Council for Evaluation of Social Development Policy; Text S1a) [27] reports a value for Nayarit close to the value for the total national population (Nayarit: 0.497 vs. Mexico: 0.516 points). Therefore, the specific objectives are four-fold: a) to test the factor structure, measurement invariance and internal consistency of the derived scales for attitudinal items of EDE-Q with CFA, considering sex and area of residence, b) to examine the convergent validity with external disordered eating questionnaire measures, c) to evaluate test-retest reliability, and d) to provide norms for the Mexican adolescent population. 

## Method

### Recruitment and Sample

Purposive sampling was used based on two criteria and considering the school as the sampling unit. The first criterion was the developmental potential justified by the *Órgano del Gobierno del Estado de Nayarit* (Government Body of the State of Nayarit) [28] under which the 20 municipalities in the state are distributed into six regions: *Norte* (North), *Sierra* (Mountain region), *Centro* (Centre), *Sur* (South), *Costa Norte* (North Coast), and *Costa Sur* (South Coast). Two municipalities were selected from each region with the exception of *Costa Norte*, which included three. The second criterion was based on demographic quantitative indicators justified by SEDESOL (Ministry of Social Development; Text S1b) [29], in combination with qualitative indicators based on social backwardness justified by CONEVAL [27]. Based on this criterion, the six regions were classified into two groups, corresponding to the level of rurality they present: urban, which includes the *Centro*, *Costa Norte*, *Costa Sur* and *Sur* regions, and rural, which includes the *Sierra* and *Norte* regions. The biggest towns (over 2,500 inhabitants) were selected from *Centro*, *Costa Norte*, *Costa Sur* and *Sur* regions, and five small villages (under 2,500 inhabitants) were selected as part of the rural subsample from *Sierra* and *Norte* regions. From the municipalities that satisfy the previous criterion, those with the most schools were selected, establishing as an inclusion criterion the fact that the schools had an Internet connection. 

Following this procedure, 36 schools were selected. Of these, one school from the *Norte* region and three from the *Centro* region refused to participate; the regional distribution is shown in Table 1. We found no statistically significant differences between the observed proportional distribution of schools by regions and the expected one considering the census information provided by INEGI (National Institute of Statistics and Geography; Text S1c) [30] [χ^2^ (5) = 9.14, *p* = .103]. For each school, the secondary and upper-secondary classes were selected using a criterion that prioritized the biggest ones. The initial sample consisted of all students present in the classroom on the day of data collection. 

**Table 1:** Sociodemographic characteristics (*N* = 2928).

		Females (*N* = 1544)	Males (*N* = 1384)
Age; *M* (SD)		15.2 (1.79)	15.0 (1.78)
Level of education/school; *n* (%)	Secondary	678 (43.9%)	691 (49.9%)
	*1^st^*	*285 (18.5%)*	*306 (22.1%)*
	*2^nd^*	*210 (13.6%)*	*211 (15.2%)*
	*3^rd^*	*112 (7.3%)*	*115 (8.3%)*
	*1^st^-3^rd a^*	*71 (4.6%)*	*59 (4.3%)*
	Upper-secondary	866 (56.1%)	693 (50.1%)
	*1^st^*	*346 (22.4%)*	*297 (21.5%)*
	*2^nd^*	*286 (18.5%)*	*223 (16.1%)*
	*3^rd^*	*234 (15.2%)*	*173 (12.5%)*
Type of school; *n* (%)	Private **^*b*^**	93 (6.0%)	56 (4.0%)
	Public	1451 (94.0%)	1328 (96.0%)
Area of residence; *n* (%)	Rural	176 (11.4%)	190 (13.7%)
	Urban	1368 (88.6%)	1194 (86.3%)
Region of origin **^*c*^**; *n* (%)	Norte/North (2/85)	57 (3.7%)	61 (4.4%)
	Sierra/Mountain region (4/91)	119 (7.7%)	129 (9.3%)
	Centro/Centre (12/156)	665 (43.1%)	651 (47.0%)
	Sur/South (5/38)	270 (17.5%)	208 (15.0%)
	Costa Norte/North Coast (5/147)	264 (17.1%)	191 (13.8%)
	Costa Sur/South Coast (4/92)	169 (10.9%)	144 (10.4%)
Socio-economic status **^*d*^**; *n* (%)	Low	506 (32.9%)	379 (27.6%)
	Medium-low	427 (27.8%)	346 (25.2%)
	Medium	277 (18.0%)	299 (21.8%)
	Medium-high	218 (14.2%)	232 (16.9%)
	High	109 (7.1%)	118 (8.6%)
Place of birth; *n* (%)	Mexico	1378 (89.6%)	1227 (88.8%)
	US	142 (9.2%)	139 (10.1%)
	Europe	18 (1.2%)	16 (1.2%)
Body mass index; *M* (SD)	(kg/m^2^)	22.52 (4.63)	22.24 (4.72)
Weight status **^*e*^**; *n* (%)	Underweight	107 (7.1%)	65 (4.8%)
	Normal weight	920 (61.1%)	832 (61.4%)
	Overweight	325 (21.6%)	307 (22.7%)
	Obesity	153 (10.2%)	151 (11.1%)

^a^ detail not available.

^b^ in Mexico the percentage of private schools is around 12% [30].

^c^ in brackets: regional distribution of the schools' sample/school's census of the municipalities selected.

^d^ based on Hollingshead’s index [32].

^e^ according to international criteria that consider sex and age [33,34] .

Based on this selection process, 3029 students participated. However, 40 students were excluded because they were older than 18 years. Of these 2989 students, *n* = 2928 adolescents aged from 11 to 18 participated in the assessment, corresponding to a response rate of 98.0% (61 were excluded because they skipped EDE-Q questions). Based on census information provided by INEGI [31], the final sample size of this study supposes 2.8% of the population of schoolchildren aged from 12 to 18 in Nayarit (*N* = 103424).

Sample characteristics are displayed in Table 1. The final study sample consisted of 1384 boys (47.3%) and 1544 girls (52.7%) with a mean age of 15.1 years (*SD* = 1.79; range 11-18 years). A follow-up sample of 505 participants (259 males and 246 females) provided two-week retest data.

Sample recruitment and data collection were conducted by the second author (AN) between 2010 and 2012. Survey administration was carried out in the schools using the paper-and-pencil method. The data were part of a larger study approved and mediated by the Nayarit State Secretary of Public Education and the Ethics Department of the Nayarit State Health Secretary, jointly with the Autonomous University of Nayarit. For all the participants, written informed consent was obtained from their families or legal guardians when appropriate.

### Measures

#### Socio-biographical data

Personal, family and social details were collected. Socioeconomic status based on the parents’ educational level and occupation according to Hollingshead’s index [32] was obtained as follows: The status score for an individual is calculated by multiplying the scale value for parental education (rated from 1 to 7) by a weight of three (3), and the scale value for parental occupation (rated from 1 to 9) by a weight of five (5). The computed scores (ranging from a high of 66 to a low of 8) are then classified into five groups of scores.

#### BMI

In situ measurements of height and weight were taken, and the BMI and weight status, according to international criteria considering sex and age [33,34], were calculated.

#### Eating Disorder Examination Questionnaire (EDE-Q) [1].

The Spanish adaptation of the EDE-Q-4 [18,20] was used. This is a 38-item self-report questionnaire that assesses attitudes, feelings and behaviours related to eating and body image over the past 28 days (available upon request). The EDE-Q generates two types of data. Firstly, 22 scaled items plus one unscaled item (Items 1-15 and 29-36) provide subscale scores reflecting the severity of aspects of the ED psychopathology. Secondly, 13 more items (Items 16-28) provide data on six key behavioural features of ED in terms of presence/absence and frequency with which the behaviour occurred, and loss of control. Lastly, the two last questions are only addressed to females (Items 37 and 38), asking about disturbances in menstruation over the past 3 months.

In the original version, four subscale scores, Restraint, Eating, Shape and Weight Concern, are theoretically derived from the average of the corresponding 22 scaled items addressing different attitudinal aspects of ED psychopathology, the response format of which is a 7-point Likert-type scale (0: *never*; 6: *everyday*). The subscale scores are obtained by calculating the average of the items forming each subscale, and the Global score is the average of the four subscale scores. A cut-off point for clinical significance of four points or more (≥ 4) for each subscale and for the global score [35], and also an empirically derived threshold of ≥ 2.30 (versus < 2.30) for the Global score [36] have been reported.

The frequency of binge eating and compensatory behaviours is assessed in terms of the average number of weekly episodes occurring during the past four weeks. Regular occurrence of these behaviours was defined as at least twice per week, based on DSM-IV-TR criteria [37]. In addition, extreme dietary restraint was calculated as “going without food for a period of eight or more waking hours in order to influence weight or shape” at an average of at least three times per week (≥ 13 days) over the past 28 days (Item 2), and extreme excessive exercise was calculated as exercising vigorously “as a means of controlling your weight, altering your shape or amount of fat, or burning off calories” at an average of at least five times per week (≥ 20 days) over the past 28 days (Item 28).

#### Children Eating Attitudes Test (ChEAT) [38].

This 26-item self-report questionnaire assesses attitudes, feelings and behaviours that are characteristic of individuals with ED, and is therefore considered a good screening tool for assessing and identifying people at risk of having an ED. It is adapted for children and is based on the original adult form [39]. Items are scored on a 6-point Likert-type scale, where "*never*", "*rarely*" or "*sometimes*" scores are equal to 0, and "*often*" is 1, "very often" is 2 and "*always*" is 3, to indicate greater severity. The Spanish adaptation we applied has shown adequate psychometric properties in Mexican children [40]. In this study, the total score was used, which showed satisfactory internal consistency (Cronbach’s α = .89).

#### Questionnaire on Influences of Aesthetic Body Ideal/*Cuestionario de Influencias del Modelo Estético Corporal* (CIMEC-26) [41]; (CIMEC-V) [42].

This 26-item self-report questionnaire evaluates the impact that different social agents (advertising, verbal messages, social models, and social situations) can have on the development of attitudes to one’s body in adolescents and young men and women aged 12-24. Participants rate items on a 3-point Likert-type scale from 0 (*never*) to 2 (*always*), and higher scores reflect a greater influence of the aesthetic body ideal. The CIMEC has shown satisfactory psychometric properties in Mexican girls [43]. In the present sample the internal consistency was satisfactory (Cronbach’s α = .93).

Eating Disorder Inventory-2 (EDI-2) [44]. This 91-item self-report questionnaire assesses cognitive and behavioural characteristics associated with ED. Items use a 6-point Likert-type scale ranging from 0 (*never*) to 5 (*always*); the three least pathological responses receive 0 points and the other responses 1, 2, and 3 to denote increasing severity. The Spanish version we applied presents good psychometric properties in Mexican samples [45]. For the present study, we used Drive for Thinness, Bulimia, and Body Dissatisfaction scales, consisting of items concerned with preoccupation with weight and dieting, tendency to binge and purge, and unhappiness with the body shape, respectively. EDI-2 was only administered to the follow-up subsample (due, in part, to its length), and acceptable internal consistency indices were obtained (Cronbach’s α values between .65 and .80).

#### Sick, Control, One, Fat, Food questionnaire (SCOFF) [46].

This is another measure used as a screening instrument for detecting ED in primary care [47]. It is a 5-item questionnaire in which participants respond with either *yes* or *no*, and the total score is the sum of positive answers. Internal consistency of this short scale in the current follow-up subsample (KR20 = .58) was similar to that found with the Spanish adaptation in a sample of young Mexican adults [48]. 

### Statistical Analysis

The statistical analyses were carried out with Mplus7 [49] and SPSS19 [50]. The 22 attitudinal items of EDE-Q were submitted to CFA with Mplus7, using maximum likelihood estimation with robust standard errors (MLR), which is a robust method for non-normality. Goodness of fit was assessed with the common fit indices [51]: χ^2^, comparative fit index (CFI), and root mean square error of approximation (RMSEA). First, four models were tested, based on previous results presented in the introduction section: a) 4-factor model (Model I) corresponding to the theoretically proposed EDE-Q subscales (Restraint, Eating concern, Shape concern, Weight concern); b) 3-factor model (Model II) that retains two EDE-Q subscales (Restraint, Eating concern) but collapses Shape and Weight concern items; c) 2-factor model (Model III) that retains one EDE-Q subscale (Restraint) but collapses Eating, Shape and Weight concern items; and d) 1-factor model (Model IV) that includes all EDE-Q subscale items and uses only the EDE-Q Global score. Second, strong measurement invariance (equal factor loadings and item intercepts) of the selected model across sex and area of residence (urban, rural) was conducted, following the common sequence for multigroup approaches [52]. For comparison of nested models, and given our large sample size, we considered that invariance should not be rejected if the decrease in CFI was lower than .01, and only a decrease in the CFI greater than .01 would be an indication of a meaningful decrement in fit [53]. And third, given the results found, the overall sample was considered with a single-sample approach; we then tested the association between EDE-Q scale scores and sex and area of residence, by adding both binary variables and their interaction as single indicators to freely correlate to the latent variable (i.e., factor). This approach involves a multiple-indicator multiple-cause (MIMIC) [54] structural equation model [55]. Therefore, the procedure permits simultaneous estimation of the measurement model and the incorporation of observed variables in the measurement model. Finally, internal consistency of the derived scales was measured through omega coefficient [56].

The other statistical analyses were performed using SPSS19. Categorical behavioural measures were compared between sex and area of residence with chi-square tests. Due to multiple comparisons, Type-I error inflation was controlled using Finner’s correction [57], obtained with SPSS macros [58]. Absolute agreement for the two-week test-retest reliability was analysed with intra-class correlation coefficients (ICCa) for quantitative measures or Cohen’s Kappa for categorical measures. Finally, Pearson’s correlation coefficients evaluated the relation between EDE-Q scores and ChEAT, CIMEC, EDI-2, and SCOFF external measures.

## Results

### Preliminary Data Analysis

Regarding the attitudinal items of EDE-Q, the percentage of missing responses was 0.06% and only 0.89% of participants showed missing values for one or more of the 22 items. Mean (and standard deviation) values ranged from 0.22 to 1.51 (0.77-2.19). Since item distributions deviated significantly from normality (median of skewness = 2.05; median of kurtosis = 3.44), the use of a robust method of estimation for CFA was supported. 

Missing data for key behavioural features were also low: 0.06% of missing responses and only 0.27% of participants showed missing values on one or more of the seven items. Mean (and standard deviation) values ranged from 0.12 to 1.18 (0.78-3.12); frequency of binge eating and compensatory behaviours were strongly positively skewed (median of skewness = 8.26) and had a high kurtosis (median of kurtosis = 98.89).

### Factor Structure, Measurement Invariance and Internal Consistency

Given the highly similar wording for three pairs of attitudinal items (6-Preoccupation with food, eating, or calories/11-Preoccupation with shape or weight; 29-Importance of weight/30-Importance of shape; and 32-Dissatisfaction with weight/33-Dissatisfaction with shape; Text S2), models were specified with these three error covariances (uniquenesses) freely estimated [59], also according to modification indices. In preliminary analyses, we compared solutions with and without these specifications to evaluate the appropriateness of this strategy [60]. The results from the CFAs (Table S1, top) showed that the three first multi-factor models provided a relatively good fit to the observed data (CFI ≥ .89, RMSEA = .054). However, model I (the original 4-factor model) yielded a non-positive definite matrix solution, which means that some variance estimates are negative, or that some exogenous variables have an estimated covariance matrix that is not positive definite. So, this model was not acceptable. Regarding model II (3-factor solution), factor correlation between Eating concern and Shape-Weight concern was .957, indicating overlap between these two factors. In contrast, factor correlation for model III was large (.784) but below .80-.85, suggesting that both factors (Restraint and Eating-Shape-Weight concern) could be distinguished [55]. Goodness-of-fit for model IV (1-factor solution) was lower (CFI = .87, RMSEA = .060). These results were unchanged when analysing each group (by sex, by area of residence and by the four groups) separately and also when analysing the four groups simultaneously (detailed results are available on request). 

We therefore selected the 2-factor model as the best model for the measurement invariance analysis across sex and area of residence (model IIIa in Table S1). Full metric invariance (equal factor loadings; model IIIb in Table S1) and full scalar invariance (equal item intercepts plus equal factor loadings; model IIIc in Table S1) across the four groups of responses was achieved, since no drop in CFI > .01 between nested models was observed. 

Internal consistency (omega coefficient) was .80 for Restraint, .92 for Eating-Shape-Weight concern, and .94 for the Global score.

### Relation to Sex and Area of Residence

Because support was found for strong (metric and scalar) measurement invariance across the four groups, indicating that none of the items showed differential item functioning, a final CFA was conducted for all participants. The inclusion of sex, area of residence and the interaction sex × area of residence as external binary indicators (MIMIC model) showed that the interaction was statistically significant for Eating-Shape-Weight concern (*p* = .003). Therefore, to represent this four-category variable, three dummy variables were included (final model in Table S1), with the males from the rural area group being used as the reference category (coded as 0). 

**Figure 1:**
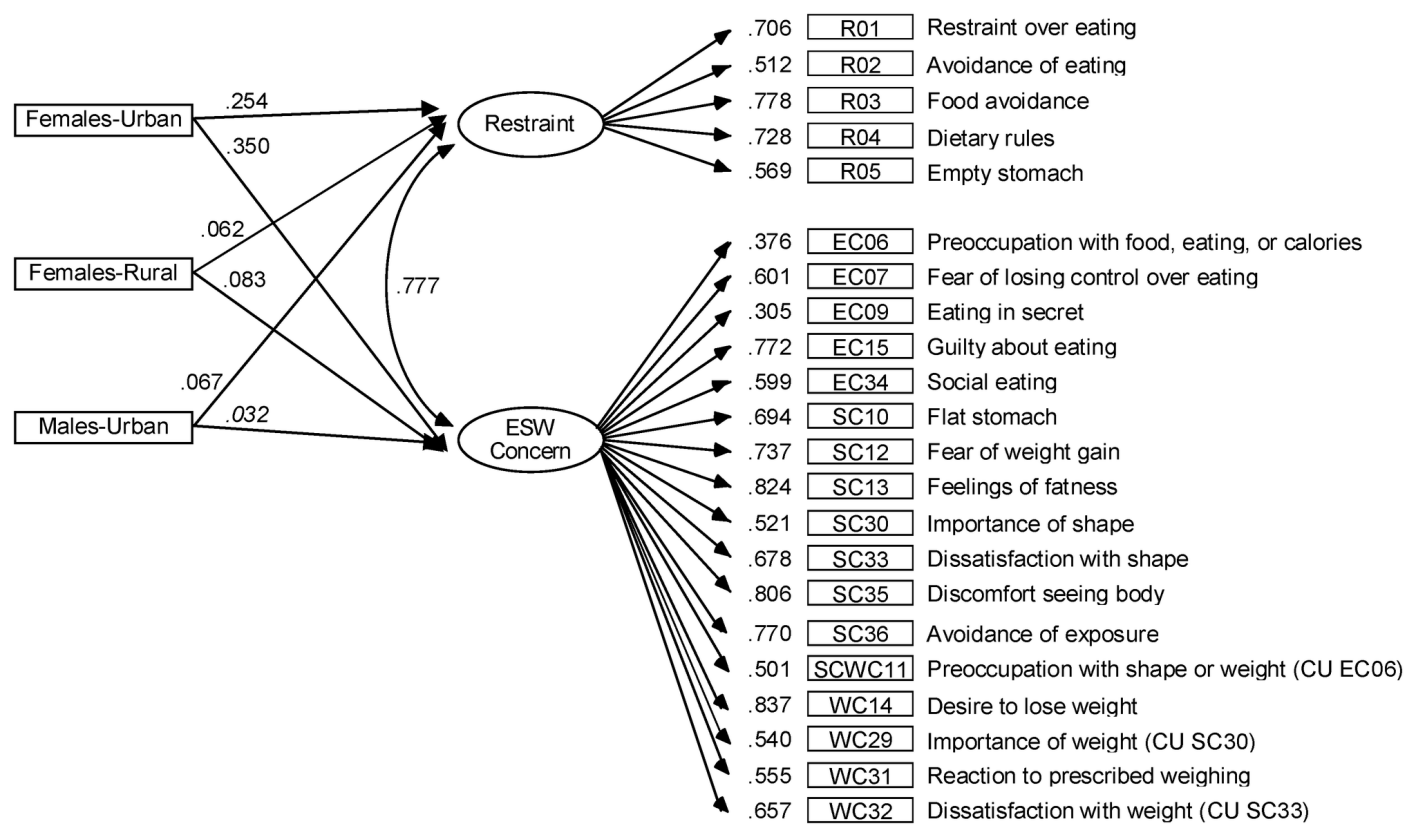
Final multiple-indicator multiple-cause (MIMIC) 2-factor model for attitudinal items of EDE-Q. Standardized parameters: factor loadings, factor correlation, and paths for factor means on sex-area groups (0: males from rural area). Item intercepts, error variances and correlated uniquenesses (CU) are omitted. In italics: parameters not statistically significant (*p* > .05). ESW denotes Eating-Shape-Weight.

Figure 1 presents factor loadings, factor correlation and paths for factor means on sex and area of residence groups for this final model. All factor loadings were statistically significant (*p* < .001) and > .30 (standardized parameters). Regarding external indicators, all parameters were positive, indicating that females from both areas of residence, especially those from urban areas, and males from urban areas scored significantly higher than males from rural areas.

### Convergent Validity

Pearson’s correlation coefficients evaluating the convergent validity between EDE-Q scale scores and external related measures were higher for females than for males (Table 2). The highest values were found for association with influence of aesthetic model (CIMEC; *r* between .75 and .43, *p* < .01), followed by drive for thinness and body dissatisfaction (EDI-2; *r* between .71 and .18, *p* < .01), eating attitudes (ChEAT; *r* between .53 and .28, *p* < .01), and the screening measure for ED (SCOFF; *r* between .64 and .25, *p* < .01) in both sexes, and bulimia (EDI-2; *r* between .38 and .34, *p* < .01) in females.

**Table 2:** Convergent validity: Pearson’s correlations between attitudinal EDE-Q scores and other related measures.

		Females			Males	
	Restraint	ESW Concern	Global Score	Restraint	ESW Concern	Global Score
ChEAT eating attitudes **^*a*^**	.48**	.50**	.53**	.28**	.33**	.34**
CIMEC influence aesthetic model **^*a*^**	.57**	.75**	.72**	.43**	.55**	.54**
EDI-DT drive for thinness **^*b*^**	.60**	.71**	.70**	.34**	.31**	.37**
EDI-BD body dissatisfaction **^*b*^**	.44**	.50**	.50**	.34**	.18**	.30**
EDI-B bulimia **^*b*^**	.34**	.38**	.38**	.11	.16*	.15*
SCOFF eating disorders’ screening **^*b*^**	.58**	.64**	.64**	.25**	.46**	.38**

ESW denotes Eating-Shape-Weight.

* *p* < .05; ** *p* < .01.

^a^ Whole sample of females (*N* = 1544) and males (*N* = 1384).

^b^ Partial follow-up subsample of females (*n* = 246) and males (*n* = 259).

### Test-retest Reliability

The two-week test-retest reliability (Table 3) was similar for both sexes, with CCIa values ranging from .84 (Restraint in males) to .90 (Global score in females) and Kappa values between .56 (any occurrence of dietary restraint in males) and .94 (extreme occurrence of dietary restraint in males).

**Table 3:** Two-week test-retest reliability of EDE-Q measures (*n* = 479-505).

	Females (*n* = 234-246)	Males (*n* = 245-259)
EDE-Q attitudinal scores		
Restraint	*.87*	*.84*
Eating-Shape-Weight Concern	*.88*	*.88*
Global Score	*.90*	*.87*
Cut-off point ≥ 2.3 (Global Score)	.79	.83
EDE-Q behavioural measures		
Any/Regular/Extreme dietary restraint	.65/.77/.67	.56/.76/.94
Any/Regular objective binge episodes	.72/.75	.73/.76
Any/Regular subjective binge episodes	.81/.75	.82/.80
Any/Regular self-induced vomiting	.84/.82	.93/.85
Any/Regular laxative misuse	.83/.79	.83/.79
Any/Regular diuretic misuse	.69/.70	.66/.71
Any/Regular/Extreme excessive exercise	.78/.76/.73	.73/.76/.67

Intraclass Correlation Coefficient (absolute agreement; ICCa) for quantitative measures (in italics);

Kappa for categorical measures (in normal font).

### Attitudinal Scores and Behavioural Measures

Table 4 shows descriptive data and percentile ranks of attitudinal derived scores for females and males from urban and rural areas. Table 4 (top) also shows the percentages for clinical significance using a cut-off point of four points or more (≥ 4) for the two subscale scores and the EDE-Q global score [35] and the empirically derived Global score threshold ≥ 2.30 [36].

**Table 4:** Percentile ranks, descriptive data, and frequency of clinically significant range for attitudinal EDE-Q scores.

		Females from urban areas (*n* = 1368)			Females from rural areas (*n* = 176)			Males from urban areas (*n* = 1194)			Males from rural areas (*n* = 190)		
		R	ESWC	GS	R	ESWC	GS	R	ESWC	GS	R	ESWC	GS
Mean		0.90 (1.18)	1.35 (1.28)	1.12 (1.14)	0.59 (0.92)	0.94 (1.07)	0.77 (0.91)	0.54 (0.90)	0.69 (0.83)	0.61 (0.78)	0.42 (0.74)	0.60 (0.76)	0.51 (0.66)
(SD)													
Cutoff point ≥ 4 (%)		3.1	5.3	3.0	1.7	3.4	1.1	1.1	1.1	0.4	0.5	0.5	0
Cutoff point ≥ 2.3 (%)		-	-	16.1	-	-	6.3	-	-	4.7	-	-	2.6
Percentile rank	5	-	0.00	0.00	-	-	-	-	-	-	-	-	-
	10	-	0.12	0.06	-	0.00	0.00	-	-	0.00	-	0.00	0.00
	15	-	0.18	0.12	-	0.06	0.03	-	0.00	0.03	-	0.04	0.03
	20	-	0.29	0.19	-	0.14	0.09	-	0.06	0.06	-	0.06	0.04
	25	-	0.35	0.26	-	0.18	0.12	-	0.12	0.09	-	0.12	0.06
	30	0.00	0.47	0.32	-	0.24	0.18	-	0.15	0.12	-	0.12	0.09
	35	0.20	0.53	0.41	-	0.35	0.22	-	0.18	0.16	-	0.18	0.10
	40	0.20	0.65	0.49	0.00	0.41	0.30	-	0.24	0.22	-	0.18	0.13
	45	0.40	0.76	0.59	0.20	0.41	0.35	0.00	0.35	0.28	-	0.24	0.19
	50	0.40	0.94	0.71	0.20	0.56	0.45	0.20	0.41	0.32	-	0.29	0.24
	55	0.60	1.08	0.84	0.20	0.71	0.53	0.20	0.47	0.38	0.00	0.36	0.28
	60	0.60	1.29	1.02	0.40	0.76	0.66	0.20	0.59	0.47	0.20	0.47	0.35
	65	0.80	1.47	1.21	0.41	0.94	0.74	0.40	0.71	0.56	0.20	0.53	0.45
	70	1.00	1.71	1.44	0.60	1.06	0.93	0.60	0.79	0.67	0.40	0.71	0.54
	75	1.40	2.12	1.66	0.80	1.28	1.04	0.60	0.94	0.82	0.60	0.82	0.67
	80	1.60	2.47	2.01	1.00	1.47	1.19	1.00	1.12	1.01	0.80	1.04	0.95
	85	2.00	2.88	2.39	1.40	2.00	1.60	1.20	1.35	1.30	1.20	1.20	1.23
	90	2.60	3.29	2.87	1.86	2.37	2.08	1.80	1.76	1.74	1.40	1.70	1.45
	95	3.60	4.06	3.51	2.60	3.48	2.66	2.60	2.47	2.26	1.89	2.17	2.17
	99	5.00	5.06	4.67	4.63	4.76	4.59	4.00	4.06	3.62	3.71	3.45	3.16
	100	6.00	5.76	5.78	5.40	4.76	4.85	6.00	5.35	5.29	4.80	4.47	3.37

R: Restraint; ESWC: Eating-Shape-Weight Concern; GS: Global Score.

Regarding key behavioural features shown in Table 5, Mexican girls endorsed significantly higher rates for dietary restraint, any subjective binging and self-induced vomiting, whereas excessive exercise was more endorsed by boys. No differences were found by area of residence (chi-square tests, *p* > .05).

**Table 5:** Frequency (%) of any, regular and extreme occurrence of key eating and compensatory behaviours and comparison between sex (*p* value for chi-square test corrected for multiple comparisons).

	Any (≥ 1/week)			Regular (≥ 2/week)			Extreme **^*a*^**		
Key behaviour (No. item)	Females	Males	*p*	Females	Males	*p*	Females	Males	*p*
Dietary restraint (it 2)	21.5	13.0	<.001	6.7	4.0	.004	4.1	1.9	.004
Objective binge episodes (it 17)	31.3	29.0	.193	19.4	16.8	.117	-	-	-
Subjective binge episodes (it 20)	17.0	13.0	.008	1.4	0.8	.171	-	-	-
Self-induced vomiting (it 22)	6.2	4.0	.014	3.9	1.7	.002	-	-	-
Laxative misuse (it 24)	4.8	3.8	.214	3.0	2.1	.171	-	-	-
Diuretic misuse (it 26)	3.9	3.9	.988	2.4	1.6	.170	-	-	-
Excessive exercise (it 28)	24.2	29.1	.008	18.1	24.1	.001	7.5	10.4	.012

^a^ Extreme dietary restraint (≥ 3/week); extreme excessive exercise (≥ 5/week).

## Discussion

This study aimed to evaluate the factor structure of the EDE-Q using CFA in the Mexican adolescent community population, considering sex and area of residence. In so doing, it also aimed to provide data on the internal structure of four alternate EDE-Q models. The current study did not provide support for the theorized 4-factor structure of the 22 attitudinal items of EDE-Q [1], a finding consistent with other studies [4-14] (also Waller and Pennings & Wojciechowski, as cited in Allen et al.) [5]. Our findings suggest that in all adolescent groups, by sex and area of residence, a 2-factor solution showed the best fit for the data, as proposed in one of the two studies previously cited that was also conducted with adolescents [7]. The notion that adolescents have difficulty in distinguishing shape and weight as separate concepts [12] seems to be also extended to Eating concern, supporting a model with a distinct Restraint subscale [7]. Therefore, only Restraint items remain as a separate dimension from Eating, Shape and Weight concern items. Tests of measurement invariance across sex and urban/rural groups also indicated that this two-factor model was stable across the four groups considered.

We think this 22-item and 2-factor model overcomes previous findings for the 22-item and 3-factor model analysed with CFA, because of the high correlation value observed for the 3-factor model between Eating concern and Shape-Weight concern, which may imply overlapping between both dimensions [55]: .96 in our case, .90 [5], or .84 [6] (values not reported in two studies) [8,9]. EFA analyses extracting 3 or 4 factors have also shown weakness, mainly in terms of crossloadings, but alternative 2-factor solutions have not been explored, despite the fact that correlations between the original subscale scores were mostly moderate [11] or high [7,12], even up to .93 [10]. Moreover, the 2-factor model we propose maintains obtaining a global score, which is aligned with the use of one general underlying dimension [4,7].

The high and significant correlations between the EDE-Q scores and the external measures of similar constructs provide further evidence of convergent validity. The pattern found was similar to that for Spanish young adult college students [18,20] and Turkish primary and high school students [21], with higher correlation values for females than males. As in previous research, correlation coefficients with EAT scores were in the range of .20-.60 [9,21,23] and the lowest values involved Restraint [13,14,21] and EDI-2 bulimia scores [18,20,24]. We also obtained satisfactory reliability indices for the EDE-Q scores, similar to or slightly higher than those in previous studies, both in terms of internal consistency [4,7,9-11,13-21] and two-week temporal stability [15,19,21,23]. 

Key behavioural features did not differ as a function of area of residence, whereas only few differences were found regarding sex [10] in the expected direction: dietary restraint, any subjective binge episode and self-induced vomiting were more endorsed by females [17,18], while excessive exercise was more endorsed by males [18], and no differences were observed for objective binge episodes [10,17], nor for the use of laxatives [10] and diuretics. These results are also aligned with findings in Mexican adolescents [61] and Mexican undergraduate students [25] using other tools than the EDE-Q. Self-induced vomiting was the more common method and diuretic misuse was the less common method. Rates of purging behaviours in this study with Mexican adolescents were slightly greater than those found in a random sample of Spanish adolescents aged 12-17 collected with the EDE-Q during 2001-2002 [17]. These differences may reflect cross-cultural differences in the expression of ED symptoms, in interpretation of the items, and/or in the availability of diuretics and laxatives. Another explanation could be that our data were collected between 2010 and 2012, and an increase in the prevalence of binge eating, purging (self-induced vomiting and/or laxative or diuretic misuse) and strict dieting or fasting for weight or shape control has been reported among both genders over a 10-year period [62]. This issue may be worrying, given that extreme weight-control behaviours seem to be the most powerful predictor of an increase in eating disturbances [63].

The highest attitudinal EDE-Q scores were found for urban females and the lowest scores were found for rural males. Rural females and urban males scored similarly (but slightly higher in the case of the former), with intermediate scores between urban females and rural males. These gender differences (females scoring higher than males) have been found in several studies [10,12,17,18,21]. In Mexican females, the attitudinal scores were lower than those reported for Restraint and the global score in Spanish female adolescents [17]. In contrast, attitudinal scores for males were very similar in both Mexican and Spanish adolescents, the former being even slightly higher. The higher Eating-Shape-Weight concern, dietary restraint, self-induced vomiting and lower excessive exercise observed in females could be explained by the fact that males are more concerned about their body shape in terms of muscularity, whereas females are more preoccupied with being thin [64].

Taken together, differences between urban and rural area of residence were only found for attitudinal scores, especially in females, but not for behavioural measures. This finding is partially aligned with the results of a previous study conducted in Mexico City, which found problematic eating attitudes and behaviours (measured with other tools than EDE-Q) between females of the same age from semi-urban and small urban towns to be quite similar [65]. These authors did not consider rural areas as we did. The rurality definition is an important point here. It has been established that there is a lack of consensus on the denotation of "rural" in educational and social work [66]. In an attempt to appraise differences in population characteristics among Mexican communities accurately, we supplemented the demographics indicators with others related to economy and infrastructure, education, and access to health. However, at the same time we were interested in excluding areas with a high likelihood of indigenous Amerindian languages in combination with a high level of social backwardness. The rationale behind this decision is related to the singularities of the living conditions in these areas, since it is not clear what impact the mass media has or which sociocultural variables are established as risk factors for the development of body dissatisfaction and eating pathologies [67]. In the end, we have established a criterion that excludes the extreme level of rurality and there are inclusion criteria (e.g., having internet access) that may limit the generalizability of results for these areas. However, we consider that a pending matter is to include Mexican adolescents from areas with a high level of rurality, to determine whether they show differences due to the impact of mass media and other sociocultural variables [67]. 

Our findings on internal structure for the 22 attitudinal items and differences across sex and area of residence led us to provide a new scoring system and normative data based on two dimensions: Restraint and Eating-Shape-Weight concern, in addition to the global score. The quality of normative data depends on the characteristics of the population sample on which they are based, in particular its size and representativeness. We have provided comprehensive normative data for the EDE-Q among Mexican adolescents, based on a large and representative sample of this population. Despite the fact that our rural groups had considerably lower sample sizes than the urban groups, we suggest the application of “rural norms” to interpret direct scores when the level of rurality of the adolescent is unknown. In so doing, one would prioritize sensitivity over specificity, because an increase in possible false positives would be more advisable in a screening scenario and/or in the first stage of a larger assessment. 

A limitation of the study is that the information concerning the adolescent population may not be generalizable to non-school adolescents from the community. A second limitation is that information regarding the ethnic diversity of the sample was not collected. Another limitation concerns the use of EDI-2 and SCOFF measures for convergent validity only in a part of the whole sample. Lastly, one school from the *Norte* region (rural area) and three schools from the *Centro* region (urban area) refused to participate, but the percentage of participating schools was similar for both areas of residence (85.7% vs. 89.7%).

Ours is the first study to date to use CFA with the EDE-Q with such a large sample [68], providing a substantial sample of urban and rural male and female Mexican adolescents, who represent nearly 3% of the population of the state of Nayarit. We can conclude that the Spanish EDE-Q shows satisfactory psychometric properties in the Mexican adolescent population. Thus, the availability of normative data for community samples would be an advantage for using EDE-Q in primary-care settings in Mexico, where practitioners may be facilitated to detect cases of ED and thus choose an appropriate referral route.

## Supporting Information

Table S1 **Goodness-of-fit indices and comparison of CFA models.**
(DOC)

Text S1 **Census information resources.** **A**. CONEVAL. **B**. SEDESOL. **C**. INEGI.
(DOC)

Text S2 **Complete content for the three pairs of items with correlated uniquenesses.**
(DOC)
