# Supplementary material for: Psychometric Properties of the Eating Disorder Examination Questionnaire (EDE-Q) and Norms for Rural and Urban Adolescent Males and Females in Mexico
Source: PLoS One. 2013 Dec 18;8(12):e83245. doi: 10.1371/journal.pone.0083245 (PMC3867461; doi:10.1371/journal.pone.0083245)
Supplement: Table S1 — Goodness-of-fit indices and comparison of CFA models. (DOC) [file pone.0083245.s001.doc]

**Table S1. Goodness-of-fit indices and comparison of CFA models.**

| Model | Goodness-of-fit indices | | | Comparison | | |
| --- | --- | --- | --- | --- | --- | --- |
|  | χ^2^ (df) | CFI | RMSEA (CI 90%) | Models | ΔCFI | Δχ^2^ (Δdf) |
| Overall sample |  |  |  |  |  |  |
| Model I: 4-factor (original: R, EC, SC, and WC) | 1884.899 (199) | .898 | .054 (.052; .056) |  |  |  |
| Model II: 3-factor (R, EC, and SC+WC) | 1909.093 (203) | .896 | .054 (.051; .056) | II *vs*. I | −.002 | 27.165 (4) |
| Model III: 2-factor (R and EC+SC+WC) | 1945.854 (205) | .894 | .054 (.052; .056) | III *vs*. I | −.004 | 59.926 (6) |
| Model IV: 1-factor (unidimensional) | 2395.979 (206) | .867 | .060 (.058; .062) | IV *vs*. I | −.031 | 362.163 (7) |
| Invariance across sex and area (4 groups) for model III |  |  |  |  |  |  |
| Model IIIa: equal form (22 items and 2 factors) | 2850.247 (820) | .873 | .058 (.056; .060) |  |  |  |
| Model IIIb: equal factor loadings (weak invariance) | 2887.616 (880) | .875 | .056 (.054; .058) | IIIb *vs*. IIIa | .002 | 84.907 (60) |
| Model IIIc: equal intercepts (strong invariance) | 3065.523 (940) | .867 | .056 (.053; .058) | IIIc *vs*. IIIb | −.008 | 162.934 (60) |
| MIMIC model (overall sample) |  |  |  |  |  |  |
| Final model (3 dummies; males-rural as reference category) | 2315.646 (265) | .893 | .051 (.049; .053) |  |  |  |

χ^2^: chi-square statistic; df: degrees of freedom; CFI: Comparative Fit Index; RMSEA: Root Mean Square Error of Approximation; CI: Confidence Interval; R: Restraint; EC: Eating Concern; SC: Shape Concern; WC: Weight Concern.
