# Supplementary material for: Psychometric Properties of the Eating Disorder Examination Questionnaire (EDE-Q) and Norms for Rural and Urban Adolescent Males and Females in Mexico
Source: PLoS One. 2013 Dec 18;8(12):e83245. doi: 10.1371/journal.pone.0083245 (PMC3867461; doi:10.1371/journal.pone.0083245)
Supplement: Text S1 — Census information resources. A. CONEVAL. B. SEDESOL. C. INEGI. (DOC) [file pone.0083245.s002.doc]

**Text S1a. CONEVAL.**

CONEVAL (National Council for the Evaluation of Social Development Policy; Spanish: Consejo Nacional de Evaluación de la Política de Desarrollo Social) is a decentralised public agency of the Federal Public Administration with technical and management autonomy, whose purpose is to coordinate the evaluation of social development policy and programs, as well as measuring poverty, according to the General Law of Social Development. CONEVAL’s Governing Body is comprised of six academic researchers from national universities and research centres, who were elected by the Federal State social development secretaries, as well as by representatives of municipalities, the Congress and the Executive Branch. The criterion for establishing social backwardness is based on indicators related to education, access to health, housing, and income.

**Text S1b. SEDESOL.**

SEDESOL (Secretariat of Social Development; Spanish: Secretaría de Desarrollo Social) is the government department in charge of social development efforts in Mexico. The Secretary of Social Development is a member of the Executive Cabinet. SEDESOL aims to eliminate poverty through comprehensive, collectively responsible human development, to achieve adequate levels of well-being with adjustment to government policies, and improvement of social, economic and political factors in rural and urban areas in order to enhance local organization, city development and housing.

**Text S1c. INEGI.**

INEGI (National Institute of Statistics and Geography; Spanish: Instituto Nacional de Estadística y Geografía) is an autonomous agency of the Mexican Government devoted to coordinating the country’s National System of Statistical and Geographical Information. It is the institution responsible for carrying out the country’s population census, economic census and agricultural, livestock and forestry census. The Institute’s task of gathering statistical information includes the monthly gross domestic product, consumer trust surveys and a proportion of commercial samples; employment and occupation statistics, domestic and couple violence; as well as many other tasks that are the basis of studies and projections carried out by other governmental institutions.
