# Supplementary material for: Psychometric Properties of the Eating Disorder Examination Questionnaire (EDE-Q) and Norms for Rural and Urban Adolescent Males and Females in Mexico
Source: PLoS One. 2013 Dec 18;8(12):e83245. doi: 10.1371/journal.pone.0083245 (PMC3867461; doi:10.1371/journal.pone.0083245)
Supplement: Text S2 — Complete content for the three pairs of items with correlated uniquenesses. (DOC) [file pone.0083245.s003.doc]

**Text S2. Complete content for the three pairs of items with correlated uniquenesses.**

Over the past 28 days...

Item 6: Has thinking about food, eating or calories made it very difficult to concentrate on things you are interested in (for example, working, following a conversation, or reading)?

Item 11: Has thinking about shape or weight made it very difficult to concentrate on things you are interested in (for example, working, following a conversation, or reading)?

Item 29: Has your weight influenced how you think about (judge) yourself as a person?

Item 30: Has your shape influenced how you think about (judge) yourself as a person?

Item 32: How dissatisfied have you been with your weight?

Item 33: How dissatisfied have you been with your shape?
